# Impact of blood glucose control on sympathetic and vagus nerve functional status in patients with type 2 diabetes mellitus

**DOI:** 10.1007/s00592-019-01393-8

**Published:** 2019-07-31

**Authors:** Yijun Yu, Liqun Hu, Yanling Xu, Shiwei Wu, Yafei Chen, Wusong Zou, Mingjing Zhang, Yuting Wang, Ye Gu

**Affiliations:** Department of Cardiology, Wuhan Fourth Hospital; Puai Hospital, Tongji Medical College, Huazhong University of Science and Technology, HanZheng Street 473#, QiaoKou District, Wuhan, 430033 China

**Keywords:** Type 2 diabetes mellitus, Glycosylated hemoglobin, Sympathetic nerve, Vagus nerve, Heart rate recovery, Heart rate variability

## Abstract

**Aims:**

Present study observed the impact of blood glucose control on sympathetic and vagus functional status in type 2 diabetes mellitus (DM) patients through observing the association between glycosylated hemoglobin (HbA1c) level and sympathetic and vagus functional status detected by heart rate recovery (HRR) and heart rate variability (HRV) assessments.

**Methods:**

Consecutive hospitalized DM patients were divided into well glycemic control group (HbA1c < 7.0%, group WGC, *n* = 100) and poor glycemic control group (HbA1c ≥ 7.0%, group PGC, *n* = 100), 100 hospitalized patients without DM served as control group (group C). All subjects underwent blood biochemistry test, treadmill exercise testing and 24-h Holter monitoring.

**Results:**

HRR and HRV parameters were significantly lower in group WGC and PGC than in group C. Standard deviation of NN intervals (SDNN), standard deviation of all 5-min average NN intervals (SDANN), very low frequency (VLF) values were significantly lower in group PGC than in group WGC. HbA1c level was negatively correlated with HRR1, SDNN, SDANN, VLF, low frequency and high frequency. Logistic regression analysis showed that lower SDNN, SDANN and VLF values were risk factors for high HbA1c levels in DM patients after adjusting for gender, age and beta-blocker use in the model 1, and for gender, age, beta-blocker use, coronary artery disease and hypertension in the model 2.

**Conclusions:**

Present results indicate that sympathetic and vagal functional status are impaired independent of HbA1c level, while poor glycemic control is related to more significant neurocardiac dysfunction in DM patients.

## Introduction

The prevalence of type 2 diabetes mellitus (DM) is increasing constantly worldwide [1]. It is known that abnormal glucose metabolism could damage major body organs such as heart, blood vessels, kidneys and nerves. Cardiovascular autonomic neuropathy (CAN) is a common complication of DM patients [2–4], and 5-year mortality rate was significantly higher in DM patients with CAN (27%) compared to DM patients without CAN (5%) [5].

CAN was traditionally evaluated by five autonomic reflex tests as described by Ewing in 1985, including: the heart rate responses to the Valsalva maneuver, standing up (30:15 ratio), and deep breathing (maximum-minimum heart rate); the blood pressure responses to standing up (postural blood pressure change), and sustained handgrip [6]. These tests are complex and require the active collaboration of patients, which limits the widespread use of this method in the daily clinical practice. There are also many other methods to evaluate cardiac autonomic regulation, including anatomic (scintigraphy), functional (muscle sympathetic nerve activity) or pharmacological (norepinephrine spillover) methodologies. Cardiac sympathetic function could be assessed by scintigraphy with ^123^I-metaiodobenzylguanidine cardiac-scintigraphy (^123^I-MIBG) and single-photon emission computed tomography (SPECT) with the development of radionuclide techniques. The nonmetabolized norepinephrine analog MIBG participates in norepinephrine uptake in postganglionic sympathetic neurons. Cardiac sympathetic function could be evaluated according the ratio of the average region of interest (ROI) in the heart (H) to the average ROI in the mediastinum (M) (the H/M ratio) in early and delayed images, and the washout rate (WR) is calculated with the formula: WR (%) = (early image H/M − late image H/M)/early image H/M × 100 [7]. The scintigraphic assessment is more sensitive in detecting CAN than indirect autonomic reflex testing [8]. However, this technology is seldom used in clinical practice because of the expensive cost. MSNA is a direct method to record sympathetic nerve activity [9]. Tungsten microelectrodes are at first inserted percutaneously into the peroneal motor tract and then the electrode should be adjusted to record the spontaneous pulse synchronized sympathetic burst activities. The mean voltage neurogram is generated through signal gain [9]. Previous study investigated the effect of pioglitazone on MSNA in DM patients with recent myocardial infarction [10]. The results showed that both of MSNA and insulin resistance index were significantly decreased after 12 weeks pioglitazone therapy, suggesting that improved insulin resistance with pioglitazone could result in the inhibition of sympathetic nerve activity in DM patients with recent myocardial infarction. However, due to the complexity of the detection method, MSNA technology is not widely used in the daily clinical setting. With the development of isotope tracing technology, total body and cardiac sympathetic activities could be estimated using the norepinephrine spillover technique [11]. Tritium norepinephrine is injected from peripheral vein for 20 min to stabilize the plasma concentration. Norepinephrine clearance and spillover rates are calculated according to a formula [11]. This technique was more accurate than catecholamine alone in assessing sympathetic neurotransmitter release. Newton et al. [12] reported that digoxin reduced cardiac norepinephrine spillover in heart failure patients with elevated filling pressures, suggesting that digoxin could reduce cardiac sympathetic nerve activity in patients with severe heart failure. The measurements could evaluate global sympathetic outflow, but they are not suitable for assessment of heterogonous changes in regional sympathetic outflow [11]. The complexity and difficulty of this method limit its clinic application. These methodologies are important for evaluating autonomic nervous system, but they are seldom used in clinic application because of poor practicability. Heart rate recovery (HRR) and heart rate variability (HRV) are derived from treadmill exercise testing and 24-h Holter monitoring, respectively, which serve as emerging indicators for evaluating autonomic nerve functional status. HRV parameters are closely related to traditional Ewing tests for the evaluation of autonomic nervous functional status in diabetes patients. Yajnik et al. [13] divided the 232 type 2 diabetes patients into three groups according to Ewing tests: normal test group (*n* = 134); one abnormal test group (*E*:*I* ratio during deep breathing or one of the two responses on standing *n* = 74); and two abnormal tests group (*E*:*I* ratio during deep breathing and one of the two responses on standing *n* = 24). They found that HRV (LF and HF) values during moderate activity were significantly lower in one and two abnormal tests groups than in normal test group, in two abnormal tests group than in one abnormal test group, indicating the close relationship between reduced HRV values and the traditional abnormal Ewing tests in diabetes patients. In recent years, HRR and HRV are widely used in clinical practice to assess cardiac sympathetic and vagus nerve functional status, due to the simple, inexpensive and non-invasive natures of these methods, which may be the valuable tools for the evaluation of CAN in DM patients [14–17].

Hypoglycemic therapy is the main approach for the prevention of organ complications in DM patients, and glycosylated hemoglobin (HbA1c) level is a common parameter measured in the clinical practice, which reflects the status of blood glucose levels in recent 3 months [18] and is also an important monitoring parameter of hypoglycemic therapy. Previous studies indicated that HRR or HRV parameters were significantly correlated with blood glucose level in DM patients [4, 19]. However, reports on the association between HbA1c level and HRR or HRV parameters in DM patients are scanty. In this study, we analyzed the association between HbA1c level and HRR/HRV parameters in type 2 DM patients and tested the hypothesis that higher HbA1c level might be linked with worse sympathetic and vagus dysfunction in DM patients.

## Subjects, materials and methods

### Study population

This retrospective study included 200 consecutive type 2 DM patients, who were hospitalized between July 2016 to December 2018 in our hospital and underwent treadmill exercise testing and 24-h Holter monitoring. Patients were divided into well glycemic control group (group WGC, HbA1c < 7.0%, *n* = 100) and poor glycemic control group (group PGC, HbA1c ≥ 7.0%, *n* = 100) according to the level of HbA1c at admission [20]. The main complaints of the hospitalized DM patients are as follows: chest pain or chest tightness (58.5%), dizziness (23.0%), polydipsia and polyuria (6.5%). Age- and gender-matched non-DM control subjects (group C, *n* = 100) were also included in this study. Type 2 DM patients were diagnosed according to the American Diabetes Association criteria [21]. Control subjects also underwent blood biochemistry test, treadmill exercise testing and obtained 24-h Holter examination. Patients with old myocardial infarction, acute coronary syndrome, complete left bundle branch block, decompensated heart failure, atrial flutter, atrial fibrillation and pacemaker implantation were excluded. The study was approved by the Ethical Committee of Wuhan Fourth Hospital, Puai Hospital affiliated to Tongji Medical College, Huazhong University of Science and Technology, Wuhan, China.

### Treadmill exercise testing and HRR analysis

Treadmill exercise testing was performed according to the exercise test guideline of the American College of Cardiology/American Heart Association [22] with GE T2100 treadmill system (General Electric Company, Boston, USA) as previously described [23]. Briefly, Bruce exercise plan was used and all subjects achieved submaximal goal heart rate [(220-age) * 0.85]. HRR1 to HRR5 for the treadmill exercise testing were calculated the difference between peak heart rate and heart rate at the 1st to 5th min of the recovery phase.

### Twenty-four-hour Holter monitoring and HRV analysis

Twenty-four-hour ambulatory electrocardiogram Holter monitoring was conducted with seer light recording box and MARS analysis software (General Electric Company, Boston, USA). HRV parameters were analyzed according to the guidelines from the European Society of Cardiology and the North American Society of Pacing and Electrophysiology [24]. The four time domain parameters included: standard deviation of NN intervals (SDNN), standard deviation of all 5-min average NN intervals (SDANN), square root of mean of the sum of squares of successive NN interval differences (rMSSD), number of successive NN interval differing by > 50 ms divided by the total number of successive NN intervals (pNN50). The four frequency domain parameters included: very low frequency (VLF) at frequency between 0.0033 and 0.04 Hz, low frequency (LF) at frequency between 0.04 and 0.15 Hz, high frequency (HF) at frequency between 0.15 and 0.4 Hz and low frequency/high frequency ratio (LF/HF).

### Statistical analysis

Continuous data were presented as mea*n* ± standard deviation. Kolmogorov–Smirnov test was performed for normal distribution of all continuous variables. Continuous variables with normal distribution among three groups were assessed with one-way ANOVA followed by Tukey’s post hoc test. Non-normal distribution variables were assessed with Kruskal–Wallis non-parametric test. Pearson’s Chi-square test was used for categorical variables as percentages. Spearman correlation analysis was performed between HRR and HRV parameters in the DM patients. The risk factors for type 2 DM were determined by multivariate logistic regression analysis. Two models were performed to adjust confounding factors. In the model 1, gender, age and beta-blockers were adjusted. In the model 2, gender, age, beta-blockers, hypertension and coronary artery disease (CAD) were adjusted. *P* value < 0.05 was considered as significant. Data were analyzed with IBM SPSS, version 22.0 for Windows.

## Results

### Comparison on clinical features among WGC, PGC and C groups

Table 1 showed the clinical characteristics of subjects in the group C, WGC and PGC. There was no significant difference on gender, age, body mass index, incidence of current smoker, CAD, hypertension, dyslipidemia and beta-blockers use, resting heart rate, peak heart rate, creatinine, cardiac troponin I (cTnI), N-terminal pro-brain natriuretic peptide (NT-proBNP) among the three groups. Fasting blood glucose and HbA1c were significantly higher in group WGC and group PGC than in group C, in group PGC than in group WGC. The high-sensitivity C-reactive protein (hs-CRP) was significantly higher in group PGC than in group WGC and group C. The incidence of diet intervention was lower, and insulin therapy was higher in group PGC than in group WGC.

**Table 1:** Clinical characteristic of group C, group WGC and group PGC

Variable	Group C (*n* = 100)	Group WGC (*n* = 100)	Group PGC (*n* = 100)
Age (yr)	59.06 ± 8.25	59.14 ± 7.58	58.74 ± 9.21
Male gender (*n*, %)	40/100 (40%)	47/100 (47%)	48/100 (48%)
BMI (kg/m^2^)	24.13 ± 3.15	24.91 ± 2.66	25.16 ± 3.45
Smoker (*n*, %)	27/100 (27%)	24/100 (24%)	21/100 (21%)
CAD (*n*, %)	32/100 (32%)	40/100 (40%)	42/100 (42%)
Hypertension (*n*, %)	70/100 (70%)	79/100 (79%)	78/100 (78%)
Dyslipidemia (*n*, %)	92/100 (92%)	90/100 (90%)	96/100 (96%)
Beta-blockers use (*n*, %)	39/100 (39%)	41/100 (41%)	49/100 (49%)
Fasting blood glucose (mM)	5.53 ± 0.98	7.16 ± 1.85**	10.32 ± 3.77**^††^
HbA1c (%)	5.58 ± 0.39	6.21 ± 0.44**	8.58 ± 1.59**^††^
Creatinine (μM)	67.51 ± 16.91	65.63 ± 16.52	62.77 ± 16.66
hs-CRP (mg/L)	2.08 ± 6.28	2.27 ± 2.48	4.83 ± 10.03**^†^
cTnI (μg/L)	0.0044 ± 0.0107	0.0041 ± 0.0053	0.0054 ± 0.0083
NT-proBNP (pM)	65.35 ± 50.22	62.20 ± 53.46	110.52 ± 314.25
Resting HR (bpm)	79.03 ± 13.05	80.74 ± 11.88	80.42 ± 10.94
Peak HR (bpm)	141.60 ± 10.05	138.91 ± 8.63	140.30 ± 8.19
Hypoglycemic therapy			
Diet intervention only (*n*, %)		33/100 (33%)	13/100 (13%)^††^
Oral anti-diabetics (*n*, %)		65/100 (65%)	69/100 (69%)
Include insulin (*n*, %)		2/100 (2%)	18/100 (18%)^††^

*Group C* control group, *Group WGC* well glycemic control group, *Group PGC* poor glycemic control group, *BMI* body mass index, *CAD* coronary artery disease, *HbA1c* glycosylated hemoglobin, *hs*-*CRP* high-sensitivity C reactive protein, *cTnI* cardiac troponin I, *NT*-*proBNP* N-terminal pro-brain natriuretic peptide, *HR* heart rate; **P < 0.01 versus Group C; ^†^*P* < 0.05, ^††^*P* < 0.01 versus Group WGC

**Fig. 1:**
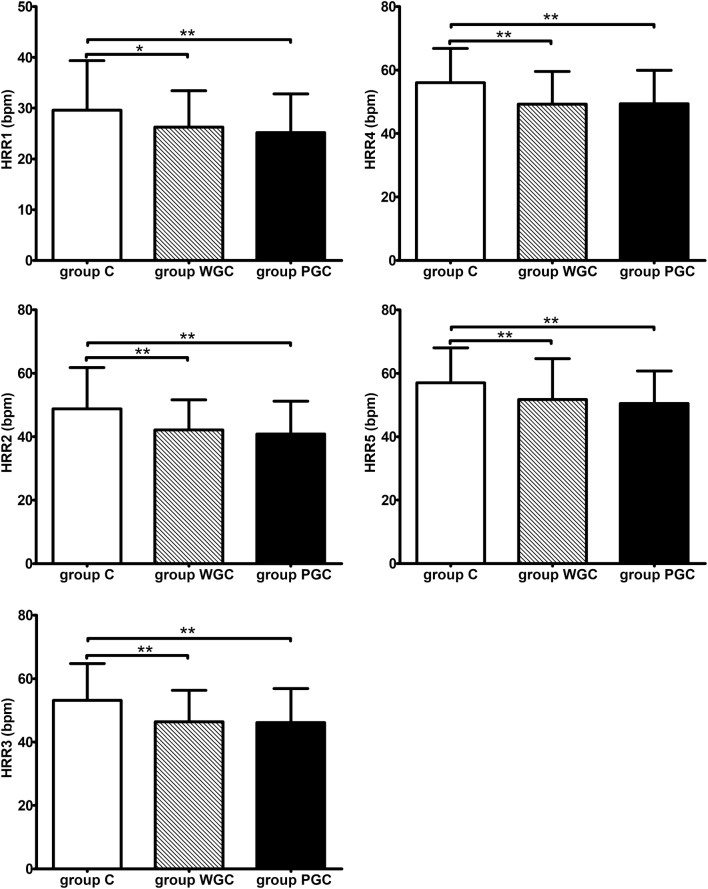
HRR analysis of group C, group WGC and group PGC. Group C, control group; Group WGC, well glycemic control group; Group PGC, poor glycemic control group; HRRn, heart rate recovery at *n* minute post-exercise; *P < 0.05; **P < 0.01

**Fig. 2:**
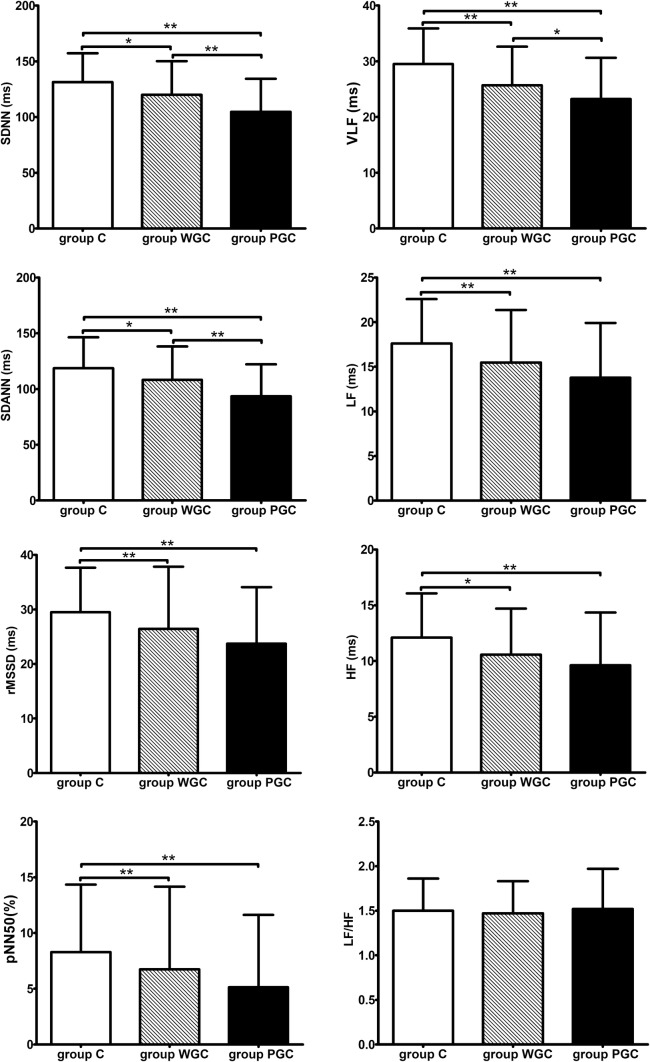
HRV analysis of group C, group WGC and group PGC. Group C, control group; Group WGC, well glycemic control group; Group PGC, poor glycemic control group; SDNN, standard deviation of NN intervals; SDANN, standard deviation of all 5-min average NN intervals; rMSSD, square root of mean of the sum of squares of successive NN interval differences; pNN50, number of successive NN interval differing by > 50 ms divided by the total number of successive NN intervals; VLF, very low frequency; LF, low frequency; HF, high frequency; *P < 0.05; **P < 0.01

Figures 1 and 2 summarized the results of HRR and HRV of various groups. HRR1 to HRR5, and HRV parameters were significantly lower in group WGC and PGC compared to group C. SDNN, SDANN, VLF values were significantly lower in group PGC than in group WGC.

### Spearman correlation analysis between HRR and HRV and between HbA1c and HRR/HRV in DM patients

Spearman correlation analysis showed that HRR1 to HRR5 were positively correlated with HRV parameters (Table 2). HbA1c level was negatively correlated with HRR1 (*r* = − 0.179, *P* = 0.011), SDNN (*r* = − 0.238, *P* = 0.001), SDANN (*r* = − 0.222, *P* = 0.002), VLF (*r* = − 0.170, *P* = 0.016), LF (*r* = − 0.171, *P* = 0.015) and HF (*r* = − 0.148, *P* = 0.036).

**Table 2:** Spearman correlation analysis between HRR and HRV parameters in DM patients

	SDNN	SDANN	rMSSD	pNN50	VLF	LF	HF	LF/HF
HRR1	0.351**	0.302**	0.310**	0.273**	0.404**	0.379**	0.353**	0.005
HRR2	0.344**	0.309**	0.276**	0.218**	0.368**	0.314**	0.347**	−0.038
HRR3	0.374**	0.347**	0.312**	0.249**	0.341**	0.293**	0.365**	−0.077
HRR4	0.388**	0.361**	0.321**	0.264**	0.328**	0.268**	0.348**	−0.101
HRR5	0.382**	0.355**	0.341**	0.278**	0.317**	0.265**	0.350**	−0.105

*HRR* heart rate recovery, *HRV* heart rate variability, *DM* diabetes mellitus, *SDNN* standard deviation of NN intervals, *SDANN* standard deviation of all 5-min average NN intervals, *rMSSD* square root of mean of the sum of squares of successive NN interval differences, *pNN50* number of successive NN interval differing by > 50 ms divided by the total number of successive NN intervals, *VLF* very low frequency, *LF* low frequency, *HF* high frequency, *HRRn* heart rate recovery at *n* minute post-exercise, **P < 0.01

### Multivariate logistic regression results for risk of DM and PGC

Multivariate logistic regression analysis demonstrated that lower HRR and HRV values were risk factors for DM (Table 3), and lower SDNN, SDANN and VLF values were risk factors for PGC among DM patients (Table 4) after adjusting for gender, age and beta-blockers (model 1) and after adjusting for gender, age, beta-blockers, hypertension and CAD (model 2).

**Table 3:** Multivariate logistic regression results for risk of autonomic nervous parameters in DM patients

Variable	Adjusted gender, age		Adjusted gender, age, CAD	
	Beta-blockers		Beta-blockers, hypertension	
	OR (95% CI)	*P* value	OR (95% CI)	*P* value
HRR1 (bpm)	1.055 (1.022–1.088)	0.001	1.052 (1.018–1.086)	0.002
HRR2 (bpm)	1.068 (1.040–1.096)	0.000	1.066 (1.037–1.095)	0.000
HRR3 (bpm)	1.066 (1.037–1.095)	0.000	1.064 (1.035–1.093)	0.000
HRR4 (bpm)	1.067 (1.038–1.096)	0.000	1.066 (1.036–1.095)	0.000
HRR5 (bpm)	1.049 (1.022–1.076)	0.000	1.047 (1.020–1.074)	0.001
SDNN (ms)	1.024 (1.014–1.034)	0.000	1.024 (1.014–1.033)	0.000
SDANN (ms)	1.021 (1.021–1.031)	0.000	1.021 (1.012–1.031)	0.000
rMSSD (ms)	1.042 (1.016–1.067)	0.001	1.043 (1.017–1.070)	0.001
pNN50 (%)	1.048 (1.012–1.087)	0.009	1.052 (1.015–1.091)	0.006
VLF (ms)	1.121 (1.076–1.166)	0.000	1.121(1.076–1.167)	0.000
LF (ms)	1.099 (1.050–1.149)	0.000	1.099(1.050–1.149)	0.000
HF (ms)	1.105 (1.043–1.171)	0.001	1.109(1.045–1.176)	0.001

*DM* diabetes mellitus, *CAD* coronary artery disease, *OR* odds ratio, *Cl* confidence interval, *HRRn* heart rate recovery at *n* minute post-exercise, *SDNN* standard deviation of NN intervals, *SDANN* standard deviation of all 5-min average NN intervals, *rMSSD* square root of mean of the sum of squares of successive NN interval differences, *pNN50* number of successive NN interval differing by > 50 ms divided by the total number of successive NN intervals, *VLF* very low frequency, *LF* low frequency, *HF* high frequency

**Table 4:** Multivariate logistic regression results for risk of autonomic nervous parameters on PGC among DM patients

Variable	Adjusted gender, age, beta-blockers		Adjusted gender, age, CAD, beta-blockers, hypertension	
	OR (95% CI)	*P* value	OR (95% CI)	*P* value
SDNN (ms)	1.018 (1.007–1.030)	0.001	1.018 (1.007–1.030)	0.001
SDANN (ms)	1.018 (1.007–1.030)	0.001	1.018 (1.007–1.030)	0.001
VLF (ms)	1.053 (1.008–1.099)	0.019	1.054 (1.009–1.100)	0.017

*PGC* poor glycaemic control group, *DM* diabetes mellitus, *CAD* coronary artery disease, *OR* odds ratio, *Cl* confidence interval, *SDNN* standard deviation of NN intervals, *SDANN* standard deviation of all 5-min average NN intervals, *VLF* very low frequency

## Discussion

Present study indicates the presence of sympathovagal imbalance in type 2 DM patients, as showed by increased sympathetic functional status (reduced SDNN, SDANN, VLF) and reduced vagal functional status (lower HRR values) in DM patients. Moreover, poor glycemic control is related to more significantly increased sympathetic functional status (lower SDNN, SDANN and VLF) in DM patients. To the best of our knowledge, this is the first clinical report assessing the sympathetic and vagal nerve functional status by combined HRR and HRV analysis and exploring their relationship in DM patients with various HbA1c level.

Previous report described delayed HRR and abnormal HRV in DM patients [2, 19]. Reduced HRR reflected impaired vagal functional status after exercise [23]. SDNN reflects total sympathetic and vagal functional status [9]. SDANN and VLF reflect sympathetic functional status [3, 25]. HF, rMSSD and pNN50 reflect vagal functional status [16]. LF reflects combined sympathetic and vagal functional status [9]. Our results indicate that neurotrophic disorders and imbalance of sympathetic and vagal nerves are common in DM patients. The underlying pathophysiological mechanism might be the interaction of following factors: abnormal glycemic metabolism, insulin resistance and compensatory hyperinsulinemia and microvascular lesions, these factors might jointly impair the autonomic nervous functional status and contribute to the development of CAN in DM patients [15]. However, the relationship between cardiac autonomic functional status and HbA1c level in DM patients is not fully understood. In this study, we found that both vagal and sympathetic nerve functional status are impaired in DM patients independent of HbA1c level. Yu and colleagues [26] found that abnormal HRR1 could predict the onset of DM, suggesting that autonomic nerve damage could occur in abnormal glucose metabolism stage before DM. These findings thus collectively imply that the impairment of vagus functional status appears early in the disease course of DM [27]. Silva et al. [28] found that lower HRV value was a risk factor for worse outcome among DM patients. Our results showed that sympathetic dysfunction is more significant in DM patients with higher HbA1c level. In line with previous finding [29], we found that hs-CRP was significantly higher in group PGC than group WGC, indicating that poor glycemic control was associated with increased inflammatory response. The incidence of insulin therapy was higher, and diet intervention was lower in group PGC than in group WGC among DM patients, indicating sympathetic dysfunction status is linked with poor glycemic control despite more frequent insulin use in DM patients. Strategies aiming to improve sympathetic functional status and reduce inflammatory response might thus be of importance in consideration of therapy plan for DM patients with higher HbA1c.

HRR and HRV parameters belong to important indicators for the evaluation of autonomic nervous functional status in patients with various diseases. HRV mainly reflects the response of autonomic nervous system to exogenous factors, while delayed HRR is considered as an indicator of decreased vagal nerve functional status. Spearman correlation analysis showed that there were close correlations between HRR and HRV, and thus, HRR and HRV might be used as complementary parameters for each other. Spearman correlation analysis also indicated that higher HbA1c level was related more severe autonomic nerve dysfunction in DM patients.

Previous studies showed that HRR or HRV values could be significantly affected by beta-blockers use, hypertension and CAD [23, 30, 31]. In our study, multivariable logistic regression analysis demonstrated that lower HRR and HRV values were risk factors for DM, and lower SDNN, SDANN and VLF values were risk factors for poor glycemic control among DM patients after adjusting for gender, age and beta-blocker use in model 1, and after adjusting for gender, age, beta-blocker use, coronary artery disease and hypertension in model 2. Therefore, the difference in HRR and HRV parameters between WGC and PGC groups was independent of beta-blockers use and comorbidities including hypertension and coronary artery disease.

### Clinical implications

This study demonstrates that delayed HRR and reduced HRV parameters, which are inexpensive and non-invasive measure of sympathetic and vagus nerve functional parameters, could be evidenced in DM patients, especially in DM patients with higher HbA1c levels. The cardiac autonomic function is impaired in DM patients, while higher HbA1c level is related more severe sympathetic dysfunction in DM patients. Therefore, HRV parameters, especially sympathetic nerve parameters including SDNN, SDANN and VLF, should be monitored in the course of hypoglycemic therapy in DM patients. Future studies are warranted to observe the impact of normalizing HbA1c in DM patients on sympathetic/vagus nerve functional status change as evaluated by HRR and HRV and the incidence of cardiovascular outcome in type 2 DM patients with high HbA1c.

### Study limitations

There were some study limitations in this study. Firstly, using HRR and HRV to evaluate autonomic nerve functional status does face significant difficulties in some patient populations. The autonomous function in patients with non-sinus rhythm such as atrial fibrillation, atrial flutter and patients with pacemaker implantation could not be adequately evaluated by HRR and HRV. Secondly, previous studies showed that exercise could improve HRR and HRV [4, 27]. In this study, lifestyle habits (occupation, exercise habit and strength, etc.) information was not obtained due to design limitation, therefore, potential impact of lifestyle habits in studied population could not be accessed, and future studies are warranted to clarify this issue. Thirdly, it is to note that our results were unfortunately not compared to the traditional method to evaluate the CAN described by Ewing et al. [6], which might enhance and validate our results, and future studies are planned to address this issue.

## Conclusions

Present study indicates that increased sympathetic while decreased vagal nerve functional status are present in type 2 DM patients independent of HbA1c level. Moreover, poor glycemic control is linked with more severely increased sympathetic functional status in DM patients. Future studies are warranted to evaluate the impact of controlling HbA1c through standardized and effective hypoglycemic therapy on vagal/sympathetic functional status and outcome in type 2 DM patients.
